# Genome-wide association studies for phenological and agronomic traits in mungbean (*Vigna radiata* L. Wilczek)

**DOI:** 10.3389/fpls.2023.1209288

**Published:** 2023-09-22

**Authors:** P. B. Manjunatha, Muraleedhar S. Aski, Gyan Prakash Mishra, Soma Gupta, Narayana Bhat Devate, Akanksha Singh, Ruchi Bansal, Shiv Kumar, Ramakrishnan Madhavan Nair, Harsh Kumar Dikshit

**Affiliations:** ^1^ Division of Genetics, ICAR- Indian Agricultural Research Institute, New Delhi, India; ^2^ Amity Institute of Organic Agriculture, Amity University, Noida, India; ^3^ Division of Plant Physiology, ICAR- Indian Agricultural Research Institute, New Delhi, India; ^4^ International Centre for Agricultural Research in the Dry Areas (ICARDA), New Delhi, India; ^5^ World Vegetable Centre, South Asia/Central Asia, Hyderabad, India

**Keywords:** mungbean, phenological traits, agronomic traits, GBS, GWAS, candidate genes

## Abstract

Mungbean (*Vigna radiata* L. Wilczek) is one of the important warm-season food legumes, contributing substantially to nutritional security and environmental sustainability. The genetic complexity of yield-associated agronomic traits in mungbean is not well understood. To dissect the genetic basis of phenological and agronomic traits, we evaluated 153 diverse mungbean genotypes for two phenological (days to heading and days to maturity) and eight agronomic traits (leaf nitrogen status using SPAD, plant height, number of primary branches, pod length, number of pods per plant, seeds per pod, 100-seed weight, and yield per plant) under two environmental conditions. A wide array of phenotypic variability was apparent among the studied genotypes for all the studied traits. The broad sense of heritability of traits ranged from 0.31 to 0.95 and 0.21 to 0.94 at the Delhi and Ludhiana locations, respectively. A total of 55,634 genome-wide single nucleotide polymorphisms (SNPs) were obtained by the genotyping-by-sequencing method, of which 15,926 SNPs were retained for genome-wide association studies (GWAS). GWAS with Bayesian information and linkage-disequilibrium iteratively nested keyway (BLINK) model identified 50 SNPs significantly associated with phenological and agronomic traits. In total, 12 SNPs were found to be significantly associated with phenological traits across environments, explaining 7%–18.5% of phenotypic variability, and 38 SNPs were significantly associated with agronomic traits, explaining 4.7%–27.6% of the phenotypic variability. The maximum number of SNPs (15) were located on chromosome 1, followed by seven SNPs each on chromosomes 2 and 8. The BLAST search identified 19 putative candidate genes that were involved in light signaling, nitrogen responses, phosphorus (P) transport and remobilization, photosynthesis, respiration, metabolic pathways, and regulating growth and development. Digital expression analysis of 19 genes revealed significantly higher expression of 12 genes, *viz*. *VRADI01G08170*, *VRADI11G09170*, *VRADI02G00450*, *VRADI01G00700*, *VRADI07G14240*, *VRADI03G06030*, *VRADI02G14230*, *VRADI08G01540*, *VRADI09G02590*, *VRADI08G00110*, *VRADI02G14240*, and *VRADI02G00430* in the roots, cotyledons, seeds, leaves, shoot apical meristems, and flowers. The identified SNPs and putative candidate genes provide valuable genetic information for fostering genomic studies and marker-assisted breeding programs that improve yield and agronomic traits in mungbean.

## Introduction

Mungbean [*Vigna radiata* (L.) R. Wilczek var. *radiata*] is an ancient grain legume cultivated extensively in South, East, and South-East Asia. Mungbean is native to Central Asia and India; however, its cultivation is rapidly expanding in other parts of the world. The area under mungbean cultivation is stretched over about 7.5–8.0 million ha globally (Nair and Schreinemachers, 2020). India is the major producer and consumer of mungbean with about 4.5 million ha area under cultivation yielding a total production of 2.5 million tons (Anonymous, 2021). Mungbean grains are an excellent source of dietary protein (22%–28%), crude fiber (4%–17%), carbohydrates, vitamins, minerals, and phytonutrients (Nair et al., 2023). In addition to providing plant-based nutrition, mungbean confers detoxification and prevention against different health ailments (Yang et al., 2020a; Kong et al., 2022). Mungbean cultivation replenishes soil nutrient status by fixing atmospheric nitrogen through symbiotic association with rhizobia, thereby reducing dependency on nitrogenous fertilizers.

Understanding the genetics and genomics of phenological and yield-associated agronomic traits, viz. grain size, grain number, pod number, and 100 seed weight are crucial to introgressing these traits to elite varieties in order to meet breeding objectives. The quantitative trait loci (QTLs) for agronomic traits have been reported in various crops such as wheat (Amalova et al., 2021; Dhakal et al., 2021; Ma et al., 2023), rice (Descalsota-Empleo et al., 2019; Tang et al., 2019; Hui et al., 2020), corn (Yang et al., 2020b), common beans (Diaz et al., 2020), and black gram (Somta et al., 2020). Several QTLs have been mapped for agronomic traits in mungbean using SSR markers (Singh et al., 2021; Kumari et al., 2022). Conventional linkage mapping uses mapping populations created through structured crosses between contrasting parents (Singh and Singh, 2015) and captures only a small portion of the phenotypic variation between the two parents. Recombination during population creation limits mapping resolution (Korte and Farlow, 2013).

GWAS offers higher mapping resolution than biparental mapping and has become a viable alternative to conventional linkage mapping. GWAS identifies QTLs based on the historic recombination that the panel of diverse germplasm has undergone through the presence of linkage disequilibrium (LD) between markers and QTLs. Hence, the power of GWAS depends on the degree of LD between the SNPs and QTLs. The resolution of genes/QTLs mapped by GWAS depends on how quickly the LD decays over a given genetic distance. The LD extent is reported as 72–290 kb in cultivated mungbean (Noble et al., 2018; Ha et al., 2021; Sandhu and Sigh, 2021) and 3–60 kb in wild mungbean (Noble et al., 2018). Technological advancements like next-generation sequencing (NGS), SNP chips, and genotyping-by-sequencing (GBS), in conjugation with the latest bioinformatics tools, further strengthen the utility of GWAS in crop improvement through marker-assisted selection. GBS allows for the discovery and genotyping of a large number of SNPs at a significantly lower cost (Elshire et al., 2011). Few studies have investigated the population structure and LD in mungbean using the GBS approach (Noble et al., 2018; Breria et al., 2020; Reddy et al., 2020; Ha et al., 2021). Genetic loci associated with mungbean seed coat color (Noble et al., 2018) and seed coat luster (Breria et al., 2020) have been identified through GWAS. Recently, 2,912 SNPs and 259 genes presence/absence variant (PAV) events associated with 33 agronomic traits were revealed by GWAS (Liu et al., 2022a). Han et al. (2022a) phenotyped 558 landraces in six distinct environments and used GWAS to identify 110 signals significantly linked to nine agronomic traits. Liu et al. (2022b) identified quantitative trait nucleotides (QTNs), QTN-by-environment interactions (QEIs), and their candidate genes for seed length (SL), seed width (SW), and 100-seed weight (HSW) in two environments using 196 accessions and 3,607,508 SNP markers. In addition, very recently, Chiteri et al. (2023) collected over 5,000 leaf images of the Iowa Mungbean Diversity (IMD) panel, consisting of 484 accessions over the course of 2 years (2020 and 2021), with two replications per experiment. Using image analysis, leaf characteristics were extracted, analyzed, and utilized in association mapping studies.

A relatively smaller genome size of 493 to 579 Mbp (Arumuganathan and Earle, 1991; Kang et al., 2014; Liu et al., 2016) makes mungbean an ideal crop for genomics study. However, despite extensive environmental, nutritional, and socioeconomic benefits, mungbean still lags behind in genomic studies due to a lack of support. The availability of only a few thousand SNP markers in mungbean is insufficient for large-scale gene mining. A draft reference mungbean genome (i.e., high-density map) of line “VC1973” from WorldVeg was constructed on the chromosome level using Illumina/Solexa and Roche 454 sequencing (Kang et al., 2014). More recently, the genome sequence of VC1973 was improved using third-generation sequencing, such as single-molecule real-time (SMRT) sequencing (Ha et al., 2021). Rapid advancements in high-throughput sequencing technologies would enable the discovery of genomic variations, strengthening modern breeding programs in mungbean.

Locus-specificity, relative abundance, co-dominant nature, and amenability to high-throughput genotyping make single nucleotide polymorphism (SNP) markers highly desirable for marker-assisted breeding (Terakami et al., 2011). The SNP markers significantly associated with agronomic traits have been reported in rice (Begum et al., 2015; Reig-Valiente et al., 2018), wheat (Rahimi et al., 2019), maize (Sadessa et al., 2022), and soybean (Kim et al., 2022). To our knowledge, limited studies have been reported for phenological and yield-associated agronomic traits in mungbean using SNP markers. In this study, we aimed to better understand the genetic diversity, population structure, LD, and genetic basis of phenological and agronomic traits in the mungbean association mapping panel representing geographically diverse regions of India, Thailand, Bhutan, Taiwan, and the Philippines. We evaluated the panel across two environments and performed GWAS to identify genomic variation for phenological and yield-associated agronomic traits.

## Materials and methods

### Association mapping panel and experimental design

The association mapping (AM) panel consisted of 153 mungbean germplasm accessions from various origins (**Supplementary Table S1**). The genotypes on the AM panel were from different countries, including India, Thailand, Bhutan, Taiwan, and the Philippines. The AM panels were planted at two locations: experimental farm, IARI, New Delhi (28° 40′ 44.6844″ N, 77° 4′ 10.9560″ E) and Punjab Agricultural University (PAU), Ludhiana (30° 54′ 3.47″ N, 75° 51′ 26.19″ E) during July 2020. Delhi (DL) and Ludhiana (LUD) are located in the Trans Ganga Plain of India and are situated at 218 msl and 247 msl, receiving an average of 886 mm and 700 mm of annual rainfall, respectively. The studied genotypes were evaluated in a randomized block design with two replications at each location (**Supplementary Table S5**). Genotypes were planted in a single row of 4 m in length, maintaining plant-to-plant spacing of 10 cm and row-to-row spacing of 30 cm. Recommended agronomic practices were followed to raise healthy crops.

### Phenotypic evaluation for phenological and agronomic traits

The panel has been characterized for phenological (days to 50% flowering, days to 100% flowering, days to maturity) and agronomic traits (leaf nitrogen status using SPAD, plant height, number of primary branches, pod length, number of pods per plant, seeds per pod, 100-seed weight, and yield per plant). Phenological traits and leaf nitrogen status using SPAD were measured in the full-bloom stage; plant architectural traits (plant height and number of primary branches) were recorded manually at maturity; and yield-associated traits (pod length, number of pods per plant, seeds per pod, 100-seed weight, and yield per plant) were recorded manually postharvest. Descriptive statistics, broad-sense heritability (*h* ^2^), and analysis of variance were carried out using Microsoft Excel. *h* ^2^ was estimated based on the ratio of genotypic to phenotypic variance (Hill and Mackay, 2004). Pearson’s correlation coefficient among the studied traits at each location was calculated and pictorially represented using the R package “corrplot” (Wei and Simko, 2017). Best linear unbiased predictors (BLUP) were calculated for each trait studied under both environments using META-R software (Alvarado et al., 2020).

### Genotyping of AM panel by GBS-based SNPs

Total genomic DNA was isolated using the CTAB method (Chen and Ronald, 1999). Out of 153 DNA samples, 126 were found to have the required quality for genotyping, and hence 126 genotypes were used for marker detection and further analysis. The samples were genotyped following a GBS approach involving complexity reduction of the genomic DNA to remove repetitive sequences prior to sequencing on NGS platforms IlluminaHiSeq 4000, as per Liu et al. (2012) and Bastien et al. (2014). The sequence data generated were aligned to the mungbean reference genome sequence using the reference-based GBS pipeline approach of STACKS v1.01 (Kang et al., 2014) to detect high-quality SNPs. SNPs obtained from GBS were imputed for missing loci with LD KNNi imputation from TASSEL v.5.0 with default parameters. Monomorphic SNPs were filtered out. SNPs with a minor allele frequency (MAF) of less than 5%, missing data of more than 10%, and heterozygote frequencies greater than 50% were also eliminated. The remaining 15,926 SNPs were used for further analysis.

### Analysis of genetic diversity and determination of population structure and linkage disequilibrium

The phylogenetic tree was constructed with 10,000 bootstraps in TASSEL using a hierarchical distance-based neighbor-joining approach. The population structure of the panel was analyzed using a clustering method based on Bayesian statistics with STRUCTURE v2.3.4 (Pritchard et al., 2003), employing 3,395 SNPs equally spaced around 50-kB distance throughout the genome. The structure was run for *K* = 1 to *K* = 10, and an ideal number of delta *K* (subpopulations) was determined using the Evanno method (Evanno et al., 2005) with Structure Harvester (http://taylor0.biology.ucla.edu/structureHarvester/). Genetic relationships among cultivated accessions were analyzed using principal component analysis (PCA) in the software package GAPIT v.3.0 (Lipka et al., 2012). The pairwise LD between SNPs was calculated based on the allele frequency correlations (*r* ^2^) using the TASSEL program v5.0 (Tao et al., 2013). The LD decay graph was drawn by fitting a smooth spline of averaged *r* ^2^ over physical distance in R v3.3.1. The LD decay was calculated when the squared correlations of allele frequencies (*r* ^2^) decreased to half of their maximum value.

### Marker trait association and digital gene expression analysis for candidate genes

Phenological and agronomic data were integrated with genome-wide SNP genotyping information generated using the association mapping panel, ancestry coefficient (*Q*), and kinship coefficient matrix (*k*) to decipher marker-trait associations. GWAS was conducted using the “BLINK” model controlling for genetic background using PCA in GAPIT v3.0 with default parameters (https://zzlab.net/GAPIT/gapit_help_document.pdf). The threshold *p-*value was set to <0.00001 to improve the overall accuracy of marker-trait associations. The SNP loci with the highest *R* ^2^ and lowest *p*-values were identified as significantly associated with phenological and agronomic traits. Identified SNP loci were compared with mungbean reference genome “*Vigna radiata* assembly v1.0” using BLAST search with “J Browse” in the “legume information system” platform (https://legacy.legumeinfo.org/genomes/jbrowse/?data=Vr1.0).

The expression pattern of the identified candidate genes was deciphered using digital gene expression analysis, where the *Arabidopsis* orthologue of the identified candidate genes from the mungbean genome was used as reference. Gene expression patterns were decoded using Expression Angler, an online search tool (Austin et al., 2016). The expression analyses were performed with several tissues, such as roots, shoots, shoot apical meristems, leaves, flowers, seeds, etc.

## Results

### Phenotypic variation of phenological and agronomic traits

**Figure 1:**
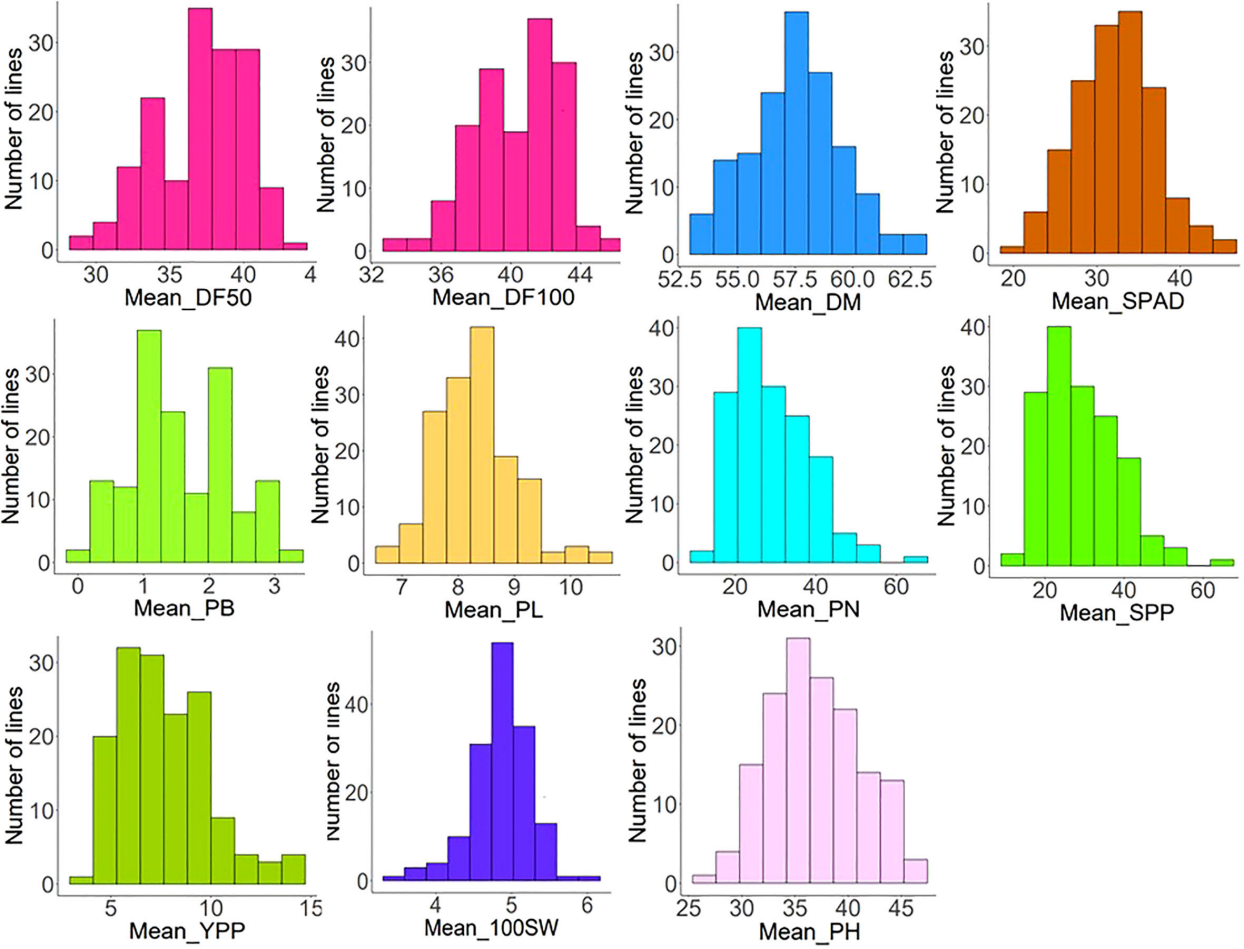
Frequency distribution patterns of phenological [days to 50% flowering (DF50), days to 100% flowering (DF100), and days to maturity (DM)] and eight agronomic traits (nitrogen status using SPAD chlorophyll meter, plant height (PH), primary branch (PB), pod length (PL), pod number (PN), seeds per pod (SPP), 100-seed weight (100SW), and yield per plant (YPP)) exhibiting normal distribution.

In two environments (Delhi and Ludhiana), 153 diverse mungbean genotypes (**Supplementary Table S1**) were evaluated for phenological and yield-associated agronomic traits. Descriptive statistics of the studied traits are presented in **Table 1**. Analysis of variance (ANOVA) revealed highly significant variation among the genotypes for all the studied traits (**Supplementary Table S2**). The interaction effect was also highly significant (*p* < 0.001) for DF50, DF100, SPAD, SPP, and YPP. Days to 50% flowering varied significantly, with an average of 37 days among early and late flowering genotypes compared across the locations (**Supplementary Table S2**). Days to maturity differed by an average of 64 days (**Supplementary Table S2**) among the genotypes. Substantial variation of yield per plant was observed, where the average minimum and maximum yield harvested across the locations were 3.47 and 14.75 g, respectively. The 100 seed weights of the genotypes varied from 2.94 g to 9.33 g across the locations. The AM panel was quite diverse in terms of the agronomic traits recorded. The coefficient of variation (CV) ranged from 2.21% to 18.22% and from 3.46% to 18.31% in the Delhi and Ludhiana environments, respectively. Traits PB and YPP showed high CV at both locations. The CV for PB and YPP were 12.12% and 18.22% in Delhi, respectively, and 18.31% and 13.51% at Ludhiana locations. The majority of the studied traits exhibited high *h* ^2^. This study showed maximum heritability for PN (0.95) followed by SPAD (0.94) at the Delhi location whereas DF50 had the highest heritability (0.94) at the Ludhiana location. The mean values for phenological traits (DF50, DF100, and DM) and yield-contributing traits, viz., PL, PN, SPP, YPP, and 100SW, were higher at the Ludhiana location compared to the Delhi location. The traits SPAD, PB, and PH were higher at the Delhi location. The frequency distribution pattern of evaluated traits showed a normal distribution, which indicates the traits are quantitative and complex in nature (**Figure 1**). Overall, sufficient phenotypic variation has been observed in the AM panel to support genome-wide association analysis.

**Table 1:** Descriptive statistics for phenological and agronomic traits studied among 153 mungbean genotypes in Delhi and Ludhiana environments.

Trait	Env	Mean ± SD	*h* ^2^	Minimum	Maximum	CV (%)	LSD	Genotype variance	Correlation
DF50	DL	35.61 ± 2.04	0.922	26.5	43	4.162	2.812	13.043^***^	0.307^***^
	LUD	38.45 ± 1.86	0.942	28.5	46.5	3.463	2.553	14.397^***^	
DF100	DL	39.25 ± 1.86	0.890	33	46	3.447	2.522	7.427^***^	0.473^***^
	LUD	41.42 ± 2.14	0.776	34.5	47.5	4.595	3.313	6.290^***^	
DM	DL	62.99 ± 1.49	0.839	50	64	2.211	2.281	4.148^***^	0.595^***^
	LUD	65.07 ± 1.91	0.552	53	66.5	3.662	3.122	2.788^***^	
SPAD	DL	32.91 ± 2.6	0.943	16.1	54.25	6.758	4.267	41.029^***^	0.304^***^
	LUD	31.38 ± 3.29	0.785	17.06	44.27	10.85	5.958	21.077^***^	
PB	DL	1.64 ± 7.22	0.459	0	4	12.12	1.145	0.310^***^	0.767^***^
	LUD	1.53 ± 7.64	0.21	0	3.5	18.31	0.241	0.011NS	
PL (cm)	DL	7.39 ± 2.25	0.900	5.45	10.05	5.077	0.703	0.635^***^	0.769^***^
	LUD	9.19 ± 2.87	0.318	7.77	10.95	8.258	0.843	0.133^*^	
PN	DL	27.41 ± 3.35	0.952	7.5	66.5	11.24	5.941	93.103^***^	0.757^***^
	LUD	30.6 ± 4.96	0.750	9.33	61	14.56	12.891	84.946^***^	
SPP	DL	10.85 ± 3.09	0.315	8.5	13.25	9.537	1.146	0.246^*^	0.696^***^
	LUD	11.35 ± 2.49	0.725	9.15	15.32	6.189	1.174	0.642^***^	
YPP (g)	DL	7.43 ± 4.27	0.814	3.47	14.75	18.22	2.412	3.981^***^	0.938^***^
	LUD	7.95 ± 3.68	0.902	4.03	14.75	13.51	2.013	5.304^***^	
100SW (g)	DL	4.57 ± 2.72	0.699	2.94	6.01	7.386	0.557	0.132^***^	0.730^***^
	LUD	5.16 ± 3.12	0.273	3.68	9.33	9.756	0.514	0.047NS	
PH (cm)	DL	37.52 ± 2.57	0.833	27.5	47.16	6.587	4.457	15.258^***^	0.809^***^
	LUD	35.87 ± 4.11	0.213	9.33	48.16	16.870	5.515	4.949NS	

Env, environment; SD, standard deviation; h2, heritability; CV, coefficient of variation; LSD, least significant difference; DF50, days to 50% flowering; DF100, days to 100% flowering; DM, days to maturity; SPAD, nitrogen status; PH, plant height; PB, primary branch; PL, pod length; PN, pod number; SPP, seeds per pods; 100SW, 100-seed weight; YPP, yield per plant. ***p < 0.001; **p < 0.01; *p < 0.05—levels of significance. NS, non significant.

### Correlation analysis

The correlation coefficient was estimated for each component trait studied in both environments (**Supplementary Figure S1**). In the Delhi location, YPP was positively correlated with PB (*r* = 0.57, *p* < 0.001), PN (*r* = 0.67, *p* < 0.001), and SPP (*r* = 0.16, *p* < 0.05). Phenological traits were positively correlated among themselves (DM and DF50, *r* = 0.44, and DM and DF100, *r* = 0.51, *p* < 0.001) as well as with PB (*p* < 0.01) and negatively correlated with plant height in both locations, i.e., Delhi and Ludhiana. The DF100 was positively correlated with the traits PL (*r* = 0.16) and PN (*r* = 0.17). The 100SW had a positive correlation with SPAD (*r* = 0.20) and PH (*r* = 0.19) (*p* < 0.05). However, a negative correlation was observed between DF100 and PH ((*r* = −0.17, *p* < 0.05) (**Supplementary Figure S1a**). Similarly, at Ludhiana, a significant positive correlation between yield with its component traits such as PB (*r* = 0.46, *p* < 0.001), PN (*r* = 0.69, *p* < 0.001), DF100 (*r* = 0.23), DM (*r* = 0.21), SPP (*r* = 0.22, *p* < 0.01), and DF50 (*r* = 0.19, *p* < 0.05) was observed. The PN was significantly correlated with DF100 (*r* = 0.29) and PB (*r* = 0.55, *p* < 0.01), DF50 (*r* = 0.17), and DM (*r* = 0.20, *p* < 0.05). Phenological traits were positively correlated, as in the case of the Delhi location, but negatively correlated with PH (*p* < 0.001) (**Supplementary Figure S1b**).

### High-throughput genotyping, genomic variant discovery, and annotation of discovered SNPs

The GBS method, employed for the genotyping of the AM panel, generated high-quality sequence reads of 264.40 million. The panel had an equal distribution of reads (mean, 1.83 million reads), and 75% of these reads on average were mapped to the *Vigna radiata* reference genome. A total of 76,160 high-quality SNPs (with read-depth 10, missing data <5%, and minor allelic frequency of 8%) were identified, out of which 55,634 chromosome-based SNPs with polymorphism were selected. The SNPs discovered were distributed throughout the genome, covering intergenic, intragenic, regulatory, and scaffold variants to the tune of 27%, 23%, 31%, and 19%, respectively. The maximum number of SNPs (2,294) were mapped on chromosome 1, whereas chromosome 10 had a minimum number of SNPs (745) mapped. Chromosome 10 had only 3.5 SNPs per 0.1 Mb, being a low SNP density (**Supplementary Table S3**). Structural annotation of 55,634 SNPs identified 25,663 (46.12%) SNPs in 11,068 protein-coding genes (intragenic region) and 29,923 (53.78%) SNPs in intergenic regions. The regulatory region harbored the largest number of gene-based SNPs (33,724 SNPs, or 60.1%), followed by the CDS region (13,030 SNPs, or 23.4%), and the intron region (10,423 SNPs, or 18.73%). A total of 5,643 missense and 7,387 synonymous SNPs, accounting for 43.4% and 56.6% of the coding SNPs, were unraveled. A SNP density plot depicting the relative distribution of GBS-based SNPs on 11 mungbean chromosomes and demonstrating variation among AM panels is presented in **Supplementary Figure S2**. The 55,634 SNP-carrying genes were functionally annotated, which prompted 2,481 associated with growth, 7,741 with development, 4,936 with metabolism, and 764 related to signal transduction proteins. Furthermore, 55,634 SNPs were imputed with TASSEL software using the LD KNNI method to remove ungenotyped markers from the haplotypes of other individuals. During imputation, SNPs were filtered to remove monomorphic markers, markers having minor allelic frequency of < 0.05 and heterozygote frequency of more than 50%. Finally, we retained 15,926 SNPs and utilized them for genetic dissections of genomic variants underlying agronomic traits. These structurally and functionally annotated SNPs may be employed for genetic dissection of QTL governing agronomic traits of mungbean.

### Analysis of genetic diversity and population structure and estimation of linkage disequilibrium

**Figure 2:**
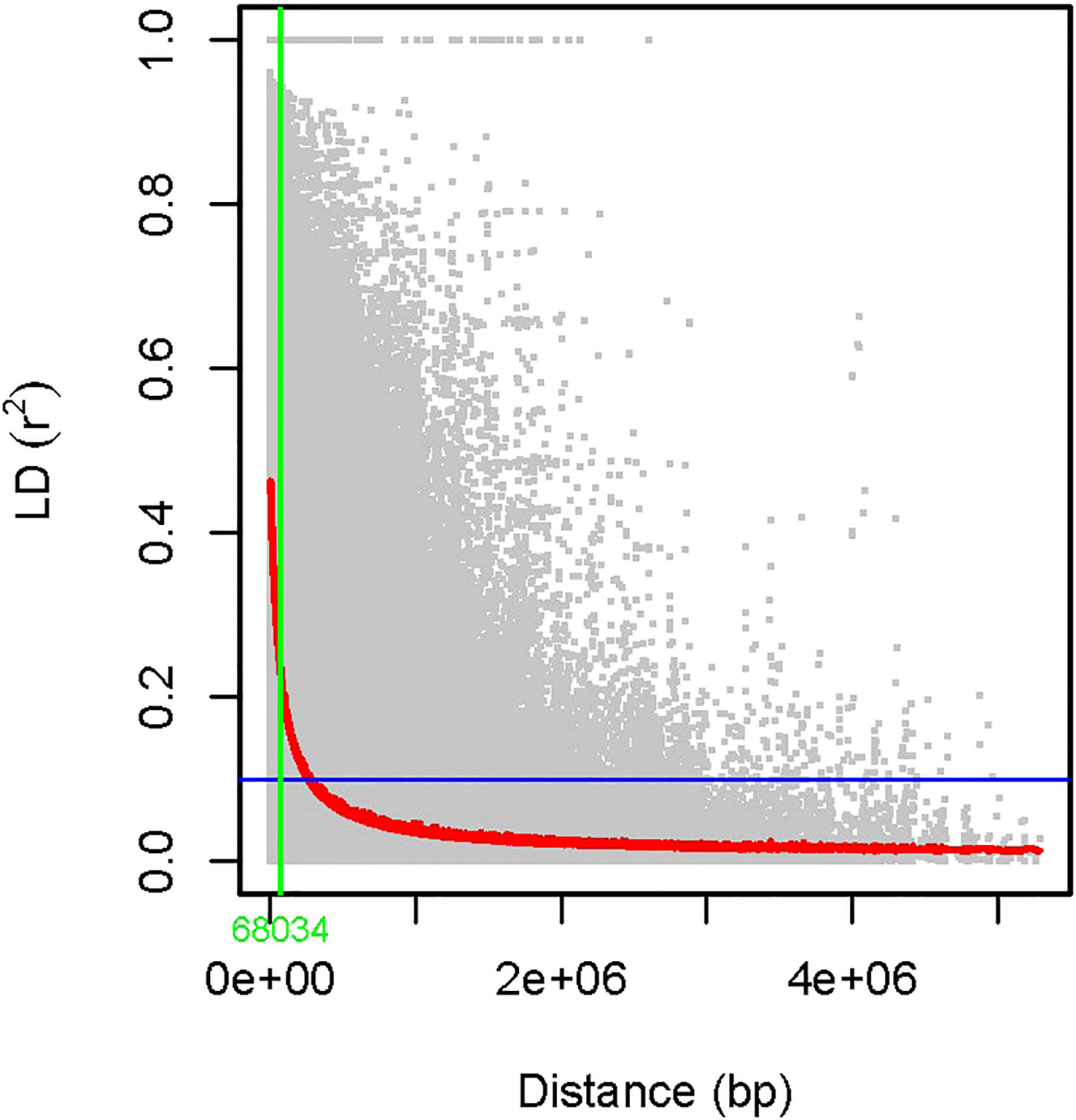
LD decay was measured in an association panel of 126 mungbean genotypes. LD was estimated between 15,926 SNP markers over the 126 mungbean accessions, and the squared correlations of allele frequencies (*r* ^2^) of the mungbean population decreased to half of its maximum value at approximately 68 kb physical distance.

The power of GWAS depends on the strength of the correlation between the marker genotype and those of the causative genes, which is a function of the distance between them. The resolution of a gene/QTL mapped by GWAS depends on how rapidly the LD decays over a particular genetic distance. In our study, linkage disequilibrium was estimated between 15,926 SNP pairs mapped on 11 mungbean chromosomes. The squared correlations of allele frequencies (*r* ^2^) of the mungbean population decreased to half of their maximum value at approximately 68 kb physical distance (**Figure 2**).

**Figure 3:**
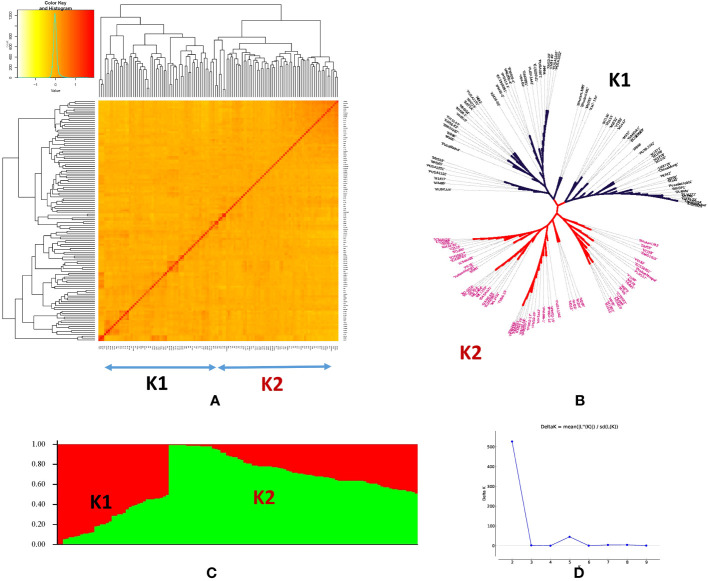
Population structure and kinship matrix similarity analysis of 126 mungbean genotypes. **(A)** Accession arrangement was based on the order of heat-map kinship similarity result. **(B)** Phylogenetic tree representing the genetic relations among 126 mungbean genotypes. **(C)** Population structure plot of the two subpopulations represented by K1 and K2 for subpopulations 1 and 2, respectively. **(D)** Delta K plot showing the best peak at *K* = 2.

**Figure 4:**
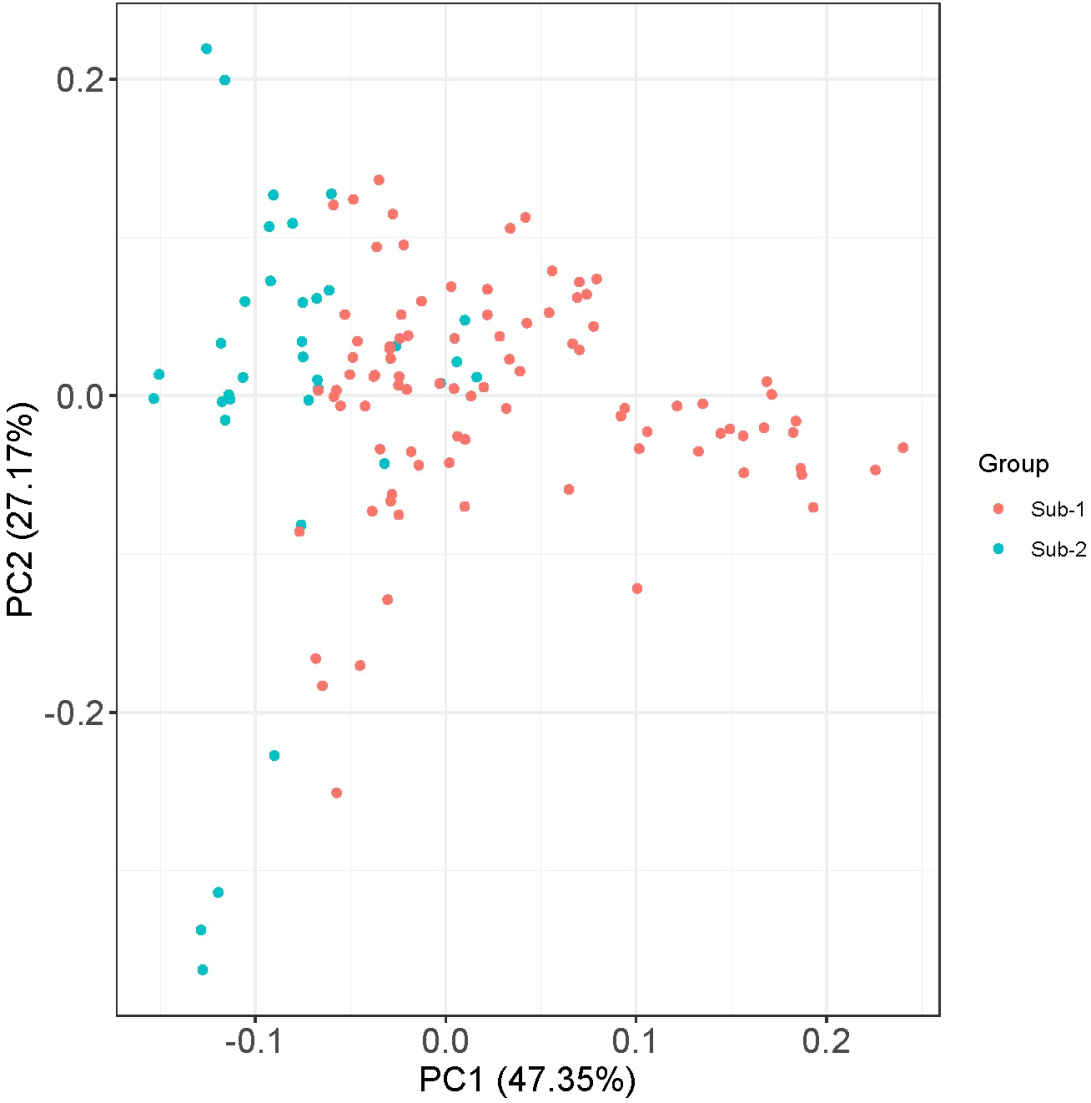
Principal component analyses (PCA) using 15,926 high-quality GBS-based SNPs assigned to 126 mungbean genotypes in two populations (1 and 2).

Population structure among the genotypes of the panel was inferred using STRUCTURE v2.3.4 software. The STRUCTURE simulations using the admixture model were run with burn-in, MCMC of 100,000, and three iterations at various levels of population size (*K* = 1 to 10). The variation in the second-order statistical rate of the Δ*K* values served as evidence for the number of hypothetical ancestral populations (*K*). The *K* value displayed a peak at *K* = 2, demonstrating the ideal number of subpopulations in the panel as two (**Figure 3**), which was validated by examining the kinship and population structure (**Figures 3A–C**). Subpopulation 1 predominantly consisted of nine germplasm lines (GL), five released varieties (RV), and one advanced breeding line (ABL), while subpopulation 2 included 11 GL, 11 RV, and seven ABL. However, the rest of the genotypes had almost equal proportions of alleles from both subpopulations and were thus classified as admixed individuals. The current panel had 82 admixture accessions. The subpopulations 1 and 2 and admixture correspond to 11.9%, 23%, and 65% of the AM panel, respectively. In subpopulation 1, GL is dominant (60%) and subpopulation 2 has been dominated by both GL and RV (75%). Furthermore, PCA was performed using a molecular marker dataset and to obtain the distance matrix among the set of genotypes, we used a matrix as input to generate a spatial representation of these genotypes in a geometric configuration as an output. The resulting multidimensional distance matrix reflecting the relationship among the set of genotypes can be represented in a 2D/3D scatter plot that validates that the current panel has two groups (i.e., two clusters) (**Figure 4**). Thus, the results from kinship, dendrogram, population structure, and PCA were in concordance.

### Marker trait associations for phenological and yield-associated agronomic traits in mungbean using the BLINK model

**Figure 5:**
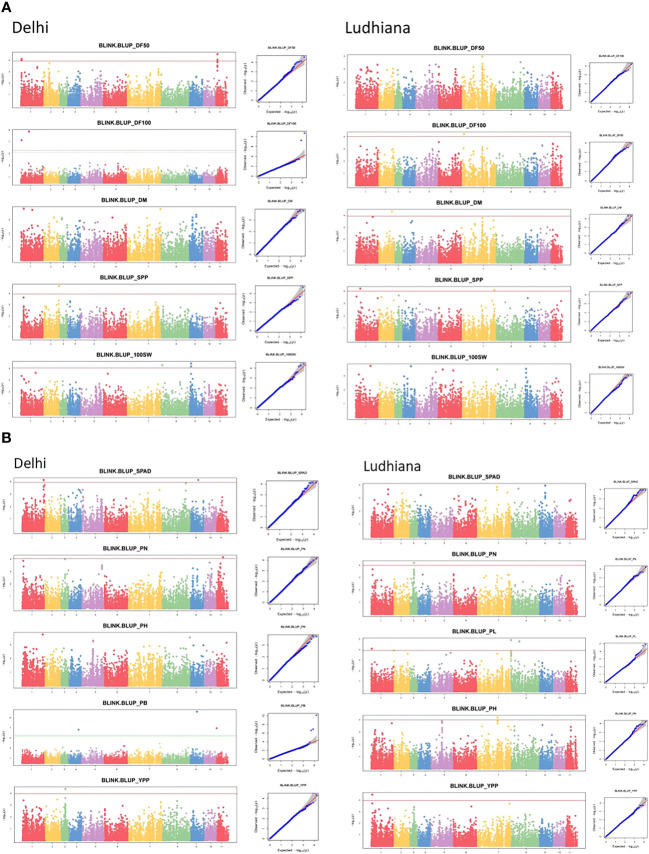
Manhattan (left) and quantile–quantile (Q-Q) (right) plots of various phenological and agronomic traits for **(A)** Delhi and **(B)** Ludhiana.

GWAS was performed using the BLINK model and PCA as a covariate in GAPIT v3.0 statistical software. Applying the threshold Bonferroni correction value of −log (*p*) = 4 as the cut-off, a total of 50 SNPs significantly associated with various phenological and agronomic traits were identified under Delhi, Ludhiana, and combined (C) BLUP environments (**Supplementary Table S4**). Marker-trait associations have been depicted in Manhattan and quantile–quantile (Q-Q) plots (**Figure 5**).

The SNPs identified by the BLINK model revealed phenotypic variation in the range of 4.7%–27.6%, 11.9%–17.2%, and 11%–20.3% in Delhi, Ludhiana, and C BLUP environments, respectively (**Supplementary Table S4**). A total of 12 SNPs were found to be significantly associated with phenological traits across environments explaining 7%–18.5% of phenotypic variation. For DF50, five SNPs were associated with the DL environment (PVE: 13.1%–16.3%). For DF100, two SNPs were significantly associated with DL (PVE: 7%–18.5%), while one SNP in LUD (PVE: 13%). Two SNPs in LUD (PVE: 11.9%) and two SNPs for C BLUP (PVE: 12.3%) were associated with DM. A total of 38 SNPs were significantly associated with yield-associated traits across environments (PVE: 4.7%–27.6%). For PL, the highest number of SNPs significantly associated under C BLUP was seven, followed by three SNPs in the LUD environment explaining 12%–16.5% of phenotypic variation. For traits PN and YPP, a single SNP was found associated with each in DL, LUD, and C BLUP environments, which explained 4.7%–17.2% and 11%–13.7% of phenotypic variations, respectively. Single SNP, each in DL and C BLUP, while two SNPs in LUD environment were associated with SPP, explaining 12.4%–14.6% of phenotypic variation for the trait. For 100 SW, three SNPs in DL, while with C BLUP, four SNPs, explaining 11%–20.3% of phenotypic variation, were identified to be significantly associated with the trait. For PB, three SNPs were detected in DL environment only, which explained 8%–27.6% of the phenotypic variation. Single SNP in LUD and two SNPs under C BLUP were associated with PH; four SNPs in DL and a single SNP in C BLUP were significantly associated with SPAD.

**Figure 6:**
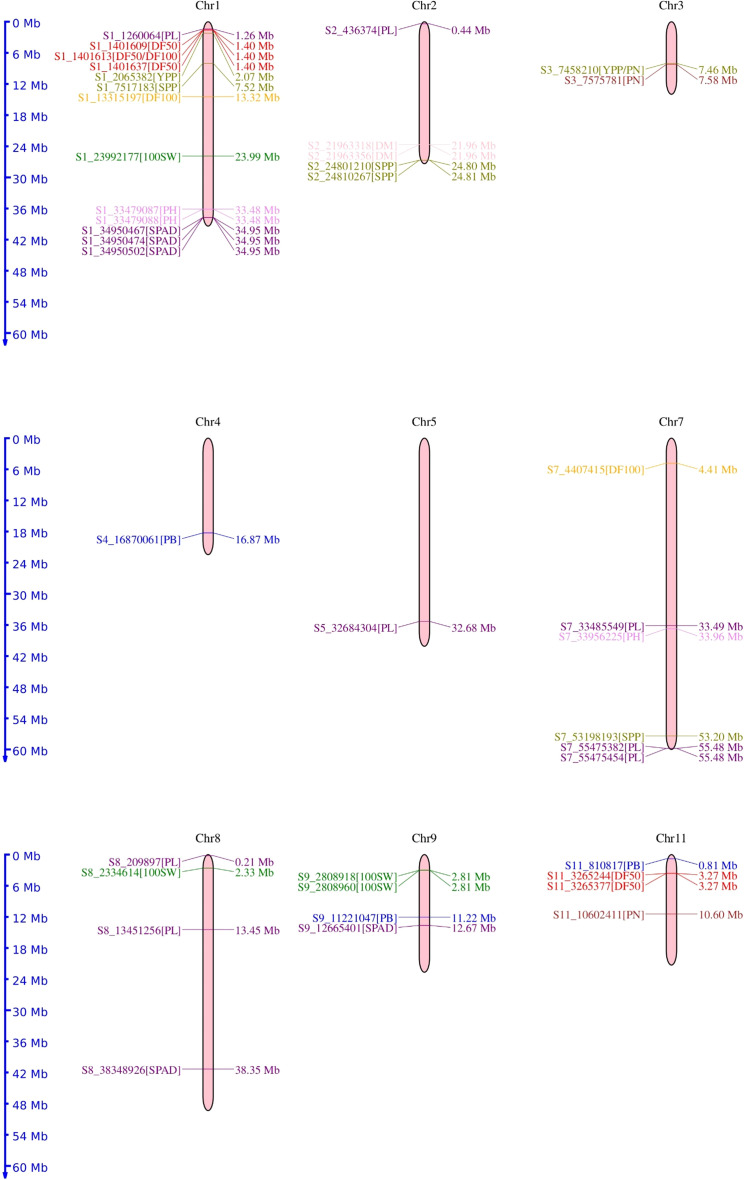
Chromosomal location of the 50 significant marker-trait associations identified for phenological and agronomic traits in mungbean genotypes.

The chromosomal distribution of SNPs revealed a maximum number of SNPs (15) to be located on chromosome 1, followed by seven SNPs each on chromosomes 2 and 8. A total of six SNPs were distributed over chromosome 6, four SNPs over chromosome 11, and three SNPs over chromosome 3. Chromosomes 4 and 5 harbored a single SNP each. However, no SNP was reported on chromosomes 6 and 10 (**Figure 6**).

### Delineation of candidate genes for phenological and agronomic traits in mungbean

Putative candidate genes for phenological and agronomic traits in mungbean were delineated by performing a BLAST search against the *Arabidopsis* genome database. The BLAST search identified 19 genes with high similarity and more than 80% identity to *Arabidopsis* genes. These identified genes were involved in the plant light signaling, nitrogen responses, P transport and remobilization, photosynthesis, respiration, multiple metabolic pathways, gametophyte development in legumes, regulating growth and development, and signaling pathways, thereby influencing yield directly or indirectly (**Table 2**). We report a total of 50 significant SNPs associated with phenological and yield-associated traits in mungbean that were found to be associated with protein-coding genes (**Figure 5**). The *Arabidopsis* orthologs of these genes are phytochrome kinase (*At1G18810*), plant regulator RWP-RK family protein (*At4G35270*), pentatricopeptide repeat (PPR) superfamily protein (*At4G14850*), cytochrome P450 superfamily protein (*At5G05690*), adenine nucleotide alpha hydrolases-like superfamily protein (*At3G53990*), calmodulin-binding family protein (*At4G33050*), subtilisin-like serine protease 2 (*At4G34980*), rhamnogalacturonatelyase family protein (*At2G22620*), carbonic anhydrase 1 (*At3G01500*), transcription factor bHLH25-like (*At4G37850*), malate dehydrogenase (*At3G47520*), phosphate-responsive 1 family protein (*At1G35140*), chaperone protein DnaJ-related (*At5G06130*), nuclear factor Y, subunit C3 (*At1G54830*), RING/FYVE/PHD zinc finger superfamily protein (*At5G22260*), receptor lectin kinase (*At3G59700*), NAD(P)H dehydrogenase (*At4G35760*), cleavage and polyadenylation specificity factor 73 kDa subunit-II (*At2G01730*), and SNARE-associated Golgi protein family (*At4G14950*). These genes might play a vital role as potential candidate genes in regulating phenological and yield-associated agronomic traits in mungbean.

**Table 2:** Putative candidate genes identified with their annotations.

Traits	ENS	SNP	*Vigna radiata* candidate gene ID	*Arabidopsis* gene orthologous ID	Protein description	Function	References
DF100	DL	S1_13315197	*VRADI01G08170*	*At1G18810*	Protein phytochrome kinase	Acts as a protein kinase in plant light signaling and thereby regulates the days to flowering	Shin et al. (2016)
SPAD	DL	S1_34950474	*VRADI01G14220*	*At4G35270*	Plant regulator RWP-RK family protein	Key regulators of nitrogen responses and gametophyte development in legumes	Chardin et al. (2014)
	DL	S1_34950467					
	DL	S1_34950502					
SPP	DL	S2_24801210	*VRADI02G14230*	*At4G14850*	Pentatricopeptide repeat (PPR) superfamily protein	Profound effects on organelle biogenesis, photosynthesis, respiration, plant development, and environmental responses	Barkan and Small (2014) and Saha et al. (2007)
SPP	DL	S2_24801210	*VRADI02G14240*	*At5G05690*	Cytochrome P450 superfamily protein	Members of this superfamily are involved in multiple metabolic pathways. Therefore, they play important roles in plant development and defense	Jun et al. (2015)
	C BLUP	S2_24810267					
YPP	DL	S3_7458210	*VRADI03G06030*	*At3G53990*	Adenine nucleotide alpha hydrolase-like superfamily protein	Plays a vital role in plant metabolism and physiology, essentially representing the major energy currency of the cell	Haferkamp et al. (2011)
100SW	DL	S8_2334614	*VRADI08G01540*	*At4G33050*	Calmodulin-binding family protein	Plays a crucial role in both regulating plant growth and development, as well as in the resistance mechanisms to various biotic and abiotic stresses in *Solanumpennellii*	Shi and Du (2020)
100SW	DL	S9_2808918	*VRADI09G02590*	*At4G34980*	Subtilisin-like serine protease 2	Broad spectrum of biological functions, ranging from plant development to interactions with the environment and plant defense responses	Figueiredo et al. (2018)
	DL	S9_2808960					
	C BLUP	S9_2808918					
	C BLUP	S9_2808960					
PN	DL	S11_10602411	*VRADI11G09170*	*At2G22620*	Rhamnogalacturonatelyase family protein	Plant development and fruit ripening, as well as its role in cell wall structure	Ochoa-Jiménez et al. (2018)
	DL	S11_10602411	*VRADI11G09180*				
PL	LUD	S1_1260064	*VRADI01G00700*	*At3G01500*	Carbonic anhydrase 1	Essential enzyme for photosynthesis and stomatal closure in plant leaves	Bhat et al. (2017)
PH	LUD	S7_33956225	*VRADI07G14230*	*At4G37850*	Transcription factor bHLH25-like	bHLH TFs are universally involved in plant growth and also play an important role in plant responses to abiotic stress in peanut	Li et al. (2021)
PH	LUD	S7_33956225	*VRADI07G14240*	*At3G47520*	Malate dehydrogenase	Plays in symbiotic nitrogen fixation, and phosphorus acquisition. Malate is the primary substrate for bacteroid respiration, thus fueling nitrogenase.	Schulze et al. (2002)
PL	C BLUP	S2_436374	*VRADI02G00450*	*At1G35140*	Phosphate-responsive 1 family protein	Playa a key role in P acquisition, transport, and remobilization in soybean	Wei et al. (2022)
PL	C BLUP	S2_436374	*VRADI02G00430*	*At5G06130*	Chaperone protein DnaJ-related	DnaJ proteins function as molecular chaperones and play critical roles in plant growth and development and response to heat stress (HS) in pepper	Fan et al. (2017)
PL	C BLUP	S5_32684304	*VRADI05G21390*	*At1G54830*	Nuclear factor Y, subunit C3	NF-Y complex plays multiple essential roles in plant growth, development, and stress responses.	Zhao et al. (2017)
PL	C BLUP	S7_55475454	*VRADI07G31390*	*At5G22260*	RING/FYVE/PHD zinc finger superfamily protein	RING zinc finger proteins have a conserved RING domain that plays important roles in plant growth, development, and responses to abiotic stresses	Han et al. (2022b)
	C BLUP	S7_55475382					
PL	C BLUP	S7_33485549	*VRADI07G14040*	*At3G59700*	Receptor lectin kinase	Plant lectin receptor kinases play crucial roles during development and in the adaptive response to various stresses.	Singh and Zimmerli (2013)
PL	C BLUP	S8_209897	*VRADI08G00110*	*At4G35760*	NAD(P)H dehydrogenase	It was identified to be essential for plant growth and development during stress periods.	Ma et al. (2021)
	LUD	S8_209897					
PL	C BLUP	S8_209897	*VRADI08G00090*	*At2G01730*	Cleavage and polyadenylation specificity factor 73 kDa subunit-II	Affects reproductive development in *Arabidopsis*	Xu et al. (2006)
	LUD	S8_209897					
SPAD	C BLUP	S8_38348926	*VRADI08G17310*	*At4G14950*	SNARE-associated Golgi protein family	SNARE proteins required for auxin-mediated development in *Arabidopsis* control many aspects of plant development.	Zhang et al. (2021)

DF50, days to 50% flowering; SPAD, nitrogen status; PH, plant height; PL, pod length; PN, pod number; SPP, seeds per pods; 100SW, 100-seed weight; YPP, yield per plant; DL, Delhi; LUD, Ludhiana; C BLUP, combined best linear unbiased predictor.

### Digital gene expression analysis of the candidate genes

The digital expression pattern of 19 genes revealed that 12 genes, namely *VRADI01G08170* (*At1G18810*), *VRADI11G09170* (*At2G22620*), *VRADI02G00450* (*At1G35140*), *VRADI01G00700* (*At3G01500*), *VRADI07G14240* (*At3G47520*), *VRADI03G06030* (*At3G53990*), *VRADI02G14230* (*At4G14850*), *VRADI08G01540* (*At4G33050*), *VRADI09G02590* (*At4G34980*), *VRADI08G00110* (*At4G35760*), *VRADI02G14240* (*At5G05690*), and *VRADI02G00430* (*At5G06130*) have high expression in different plant parts, including roots, cotyledons, seeds, leaves, shoot apical meristems, and flowers are depicted in **Supplementary Figure S3**.

## Discussion

### Phenotypic evaluation and characterization

Evaluation and characterization of yield-contributing traits are prerequisites in any crop improvement program. Evaluation and characterization lead to the identification of superior genotype(s) and equip breeders with the most vital input for successful crop breeding. Nearly 1,500 indigenous and exotic collections were characterized and evaluated for 27 agro-morphological traits in mungbean. The study revealed that the cultivated mungbean gene pool exhibits a higher degree of variation for economically important traits like seed weight, flowering period, pod length, number of seeds per pod, and seed size (Gayacharan et al., 2020). Similarly, the World Vegetable Centre, Taiwan, has characterized and evaluated global mungbean collections of 5,234 accessions for eight agro-morphological traits, including primary leaf width, primary leaf length, days to 50% flowering, plant height at flowering, plant height at maturity, pod length, seeds per pod, and 1,000 seed weight. A wide array of phenotypic variability was evident for certain characters in these global mungbean accessions, which was expressed in terms of Shanon’s diversity index of 0.82 (average of all traits) (Schafleitner et al., 2015). In line with the above studies, we characterized 153 mungbean genotypes at Delhi and Ludhiana for phenological and various yield-associated agronomic traits, including SPAD, PH, PB, PL, PN, SPP, 100SW, and YPP. The ANOVA unveiled the presence of highly significant variation among evaluated genotypes. Phenotypic variability in the present study could be attributed to a large number of genotypes belonging to different eco-geographical regions of the world. Significantly large phenotypic variability for agronomically important traits in the present study is in agreement with previously published reports (Reig-Valiente et al., 2018; Khan et al., 2022). Kumari et al. (2022) evaluated wild and cultivated *Vigna* species for phenological (days to first flowering and days to first pod maturity) and other agronomic traits (plant height, pod length, number of seeds per plant, and 100-seed weight). Traits with high heritability (pod number, SPAD, YPP, DF100, and DF50) may be successfully deployed in breeding programs.

### Correlation study among agronomic traits

Correlation studies among the best combinations of attributes significantly contributing to yield give insight into traits that are inherited together. High trait correlations may be due to the direct or indirect contribution of attributes towards one or more traits or because of pleiotropic effects. Positive correlation among traits of interest makes crop improvement easy and effective. Recognizing the importance of correlation among yield-contributing traits, many researchers have studied the correlation among yield traits in different crops (Mwadzingeni et al., 2017; Li et al., 2018; Rahimi et al., 2019). In the present study, yield per plant was positively correlated with PB, PN, and SPP. Phenological traits were positively correlated among themselves as well as with PB. The DF100 was positively correlated with traits PL and PN. However, a negative correlation was observed between DF100 and PH. Similarly, at Ludhiana, a significant positive correlation between yield and its component traits (PB, PN, DF100, DM, SPP, and DF50) was observed. Phenological traits were positively correlated, as in the case of the Delhi location, but negatively correlated with PH. A positive correlation between heading date and grain number per panicle was reported by Li et al. (2018). Rahimi et al. (2019) reported a positive association between yield and its component traits such as the number of seeds per spike, thousand kernel weight, and grain filling period. They found yield to be negatively correlated with phenology traits. Contreras-Soto et al. (2017) found a positive correlation between seed yield and seed weight in soybean.

### LD in mungbean is comparable to closely related species

The power of the GWAS approach depends on the degree of LD between SNP and QTL, which is determined by the genetic distance between them. In addition, the resolution of a gene/QTL mapped by GWAS and the density of marker coverage needed for GWAS depend on how rapidly the LD decays over a particular distance (Myles et al., 2009). If LD decays faster than expected, a higher marker density is required to capture markers associated with causal loci (Lin et al., 2019). In the current study, the genome-wide LD decayed at genomic distances of about 68 kb. Previous studies revealed that the LD extent is about 72–290 kb in cultivated mungbean (Noble et al., 2018; Ha et al.,2021; Sandhu and Sigh, 2021) and 3–60 kb in wild mungbean (Noble et al., 2018). LD pattern in mungbean was determined to be distinct from chickpea (Saxena et al., 2014; Kujur et al., 2016) but presumably similar to other self-pollinated crop species, such as soybean (Patil et al., 2016).

### High-resolution association mapping study

GWAS makes use of ancient recombination events that occurred in a group of genotypes, mainly in landraces, to identify either a causative gene for the trait of interest or to unravel the genetic architecture of complex traits by finding DNA markers, usually SNPs, underlying particular traits at relatively high resolution. The completion of the mungbean reference genome sequence by Kang et al. (2014) allowed the possibility of using GWAS in mungbean. GWAS using GBS has been employed in several studies to examine population structure in mungbean (Noble et al., 2018; Breria et al., 2020; Ha et al., 2021). Very recently, GWAS in mungbean identified 2,912 SNPs and 259 gene PAV events underlying 33 agronomic characteristics (Liu et al., 2022a). GWAS in mungbean landraces phenotyped in six different environments led to the identification of 110 signals significantly associated with nine agronomic traits (Han et al., 2022a). Liu et al. (2022b) identified QTNs, QEIs, and their candidate genes for SL, seed width, and HSW in two environments using 196 diverse accessions genotyped with 3,607,508 SNP markers. Recently, Chiteri et al. (2023) collected over 5,000 leaf images of the IMD panel, consisting of 484 accessions over 2 years having two replications per experiment. Leaf traits were extracted using image analysis, analyzed, and used for association mapping studies.

In the present study, GWAS with the BLINK model revealed 50 novel SNPs that were shared by DL, LUD, and CBLUP environments. These genome-wide distributed SNP markers have been found to be associated with three phenological (DF50, DF100, and DM) and eight agronomic traits, viz., SPAD, PH, PB, PL, PN, SPP, 100SW, and YPP. Sandhu and Singh (2021) carried out GWAS for agronomic traits (days to flowering, plant height, and seed size) in a USDA mungbean collection of 482 accessions using 264,550 SNPs and discovered three SNP loci on different chromosomes for each trait. Of the 50 reported genes, 19 were found to play diverse roles in stress, growth, and developmental pathways. This study could identify genes containing RWP-RK family protein, PPR superfamily protein, cytochrome P450 superfamily protein, adenine nucleotide alpha hydrolases-like superfamily protein, calmodulin-binding family protein, rhamnogalacturonatelyase family protein, phosphate-responsive 1 family protein, DnaJ-related chaperone protein, RING/FYVE/PHD zinc finger superfamily protein, and SNARE-associated Golgi protein family, and also enzymes like protein phytochrome kinase, subtilisin-like serine protease 2, carbonic anhydrase 1, malate dehydrogenase receptor lectin kinase, and NAD(P)H dehydrogenase. These family and enzyme-containing genes have been reported to play a diverse role in controlling the growth and development signals of crop plants. Days to flowering are pivotal traits responsible for adaptation, and it showed maximum sensitivity to environmental photoperiod and temperature in various crops (Lu et al., 2020). The present study reported that the gene *VRADI01G08170* encodes a phytochrome kinase enzyme that acts in plant light signaling and thereby regulates the days to flowering (Shin et al., 2016). Leaf nitrogen content per leaf area and SPAD readings are highly affected by environmental factors (Xiong et al., 2015). In the current study, the gene *VRADI01G14220* encodes the RWP-RK family protein, a key regulator of nitrogen responses and gametophyte development in legumes (Chardin et al., 2014). The gene *VRADI07G14240* encodes the malate dehydrogenase enzyme that acts in symbiotic nitrogen fixation and phosphorus acquisition (Schulze et al., 2002). Similarly, the gene *VRADI02G00450* is a phosphate-responsive 1 family protein playing a key role in P acquisition, transport, and remobilization in soybeans (Wei et al., 2022). As a result, these candidate genes may regulate nutrient uptake and contribute to mungbean development.

A PPR encoded by the gene *VRADI02G14230* plays a key role in photosynthesis and respiration (Saha et al., 2007; Barkan and Small, 2014). Similarly, the gene *VRADI01G00700* governs the carbonic anhydrase 1 enzyme, which regulates photosynthesis and stomatal closure in plant leaves (Bhat et al., 2017). Therefore, higher photosynthetic rates and stomatal density are necessary for indirect regulation of important economic traits such as pod length, pod number, seeds per pod, and plant architecture. The gene *VRADI11G09170* encodes the rhamnogalacturonatelyase family protein involved in fruit/pod ripening (Ochoa-Jiménez et al., 2018). Thus, it may promote early pod maturity in mungbean.

Furthermore, the gene *VRADI09G02590* encodes subtilisin-like serine protease 2 enzymes, which help in plant development and interactions with the environment (Figueiredo et al., 2018), which may help to adapt to diverse environmental conditions. Similarly, candidate genes such as *VRADI02G14240*, *VRADI03G06030*, *VRADI08G01540*, *VRADI07G14230*, *VRADI02G00430*, *VRADI05G21390*, *VRADI07G31390*, *VRADI07G14040*, *VRADI08G00110*, *VRADI08G00090*, and *VRADI08G17310* are involved in regulation of plant growth and development during various stages (Xu et al., 2006; Haferkamp et al., 2011; Singh and Zimmerli, 2013; Jun et al., 2015; Fan et al., 2017; Zhao et al., 2017; Shi and Du, 2020; Li et al., 2021; Ma et al., 2021; Zhang et al., 2021; Han et al., 2022b). Sandhu and Singh (2021) reported a SNP 1_11367629 located within LOC106774729, which produces the receptor-like protein kinase FERONIA (FER). FER has diverse functions in plant growth and development. As a consequence, these candidate genes may play both direct and indirect roles in regulating important agronomic traits in mungbean.

Additionally, digital expression analysis of 19 genes revealed significantly higher expression of 12 genes, viz. *VRADI01G08170* (*At1G18810*), *VRADI11G09170* (*At2G22620*), *VRADI02G00450* (*At1G35140*), *VRADI01G00700* (*At3G01500*), *VRADI07G14240* (*At3G47520*), *VRADI03G06030* (*At3G53990*), *VRADI02G14230* (*At4G14850*), *VRADI08G01540* (*At4G33050*), *VRADI09G02590* (*At4G34980*), *VRADI08G00110* (*At4G35760*), *VRADI02G14240* (*At5G05690*), and *VRADI02G00430* (*At5G06130*) in the roots, cotyledons, seeds, leaves, shoot apical meristems, cotyledons, dry seeds, and flowers. The candidate genes identified in the present study may be involved in regulating phenological and yield-associated agronomic traits in mungbean. So far, only a few studies have been carried out for agronomic traits employing GWAS in mungbean. Hence, it is difficult to pinpoint precisely which candidate gene(s) significantly regulates agronomic traits in mungbean, which calls up for further in-depth study. The SNP information linked with distinct candidate genes found in GWAS analysis needs further validation, either in diverse populations or by using laboratory tests such as overexpression and knockout of candidate genes. Furthermore, true causal genes can be effectively deployed for developing superior cultivars in mungbean through MAS.

## Data availability statement

Original SNP datasets are available in a publicly accessible repository: The original contributions presented in the study are publicly available. This data can be found here: [https://www.ncbi.nlm.nih.gov/sra/PRJNA609409.]. Phenotyping data used in this study is available in **Supplementary Material**.

## Author contributions

PM: conceptualization, investigation, data curation, and writing original draft. MA: methodology, software, and investigation. GM: conceptualization, resources, and writing—review and editing. SG: formal analysis and writing—review and editing. ND: investigation and formal analysis. AS: formal analysis. RB: methodology. SK: conceptualization and writing—review editing. RN: writing—review editing and fund acquisition. HD: conceptualization, methodology, writing—review editing, supervision, project administration, and fund acquisition. All authors contributed to the article and approved the submitted version.
