# Supplementary material for: Genome-wide association studies for phenological and agronomic traits in mungbean (Vigna radiata L. Wilczek)
Source: Front Plant Sci. 2023 Sep 22;14:1209288. doi: 10.3389/fpls.2023.1209288 (PMC10558178; doi:10.3389/fpls.2023.1209288)
Supplement: Supplementary file 1 [file DataSheet_1.pdf]

Supplementary Table 1. List of 153 mungbean genotypes along with their source.

Sr. No.	Genotype	Type	Source/Origin
1	BASANTI	RV	Haryana, India
2	BhutanLM1	RV	Bhutan
3	BhutanLM2	RV	Bhutan
4	BhutanLM95	RV	Bhutan
5	ChinaMung	RV	China
6	DMS10	RV	Bihar, India
7	DMS4	RV	Bihar, India
8	DMS8	ABL	Bihar, India
9	EC3988891	GL	AVRDC, Thailand
10	EC520024	GL	AVRDC, Thailand
11	EC520029	GL	AVRDC, Thailand
12	EC520041	GL	AVRDC, Thailand
13	EC550851	GL	AVRDC, Thailand
14	GANGA1	RV	Rajasthan, India
15	GANGA8	RV	Rajasthan, India
16	HUM1	RV	Uttar Pradesh, India
17	HUM16	RV	Uttar Pradesh, India
18	HUM2	RV	Uttar Pradesh, India
19	HUM6	RV	Uttar Pradesh, India
20	IC28083	GL	New Delhi, India
21	IC282094	GL	New Delhi, India
22	IC325828	GL	New Delhi, India
23	IC436636	GL	New Delhi, India
24	IC436637	GL	New Delhi, India
25	IC436763	GL	New Delhi, India
26	IC546476	GL	New Delhi, India
27	IC546488	GL	New Delhi, India
28	IPM02-14	RV	Uttar Pradesh, India
29	IPM02-15	ABL	Uttar Pradesh, India
30	IPM02-17	ABL	Uttar Pradesh, India
31	IPM02-19	ABL	Uttar Pradesh, India
32	IPM02-3	RV	Uttar Pradesh, India
33	IPM02-30	ABL	Uttar Pradesh, India
34	IPM205-4	ABL	Uttar Pradesh, India
35	IPM205-7	RV	Uttar Pradesh, India
36	IPM288	ABL	Uttar Pradesh, India
37	IPM406-1	ABL	Uttar Pradesh, India
38	IPM409-4	ABL	Uttar Pradesh, India
39	IPM410-3	RV	Uttar Pradesh, India
40	KM11-40	ABL	Uttar Pradesh, India
41	KM12-28	ABL	Uttar Pradesh, India
42	KM16-18	ABL	Uttar Pradesh, India

43	KM16-23	ABL	Uttar Pradesh, India
44	KM16-50	ABL	Uttar Pradesh, India
45	KM16-60	ABL	Uttar Pradesh, India
46	KM16-69	ABL	Uttar Pradesh, India
47	KM16-75	ABL	Uttar Pradesh, India
48	KM16-80	ABL	Uttar Pradesh, India
49	KM16-82	ABL	Uttar Pradesh, India
50	KM2241	ABL	Uttar Pradesh, India
51	KM7-134	ABL	Uttar Pradesh, India
52	LGG460	RV	Andrapradesh, India
53	M1032	GL	AVRDC, Taiwan
54	M1053	GL	AVRDC, Taiwan
55	M1129	GL	AVRDC, Taiwan
56	M1131	GL	AVRDC, Taiwan
57	M1156	GL	AVRDC, Taiwan
58	M1168	GL	AVRDC, Taiwan
59	M1209	GL	AVRDC, Taiwan
60	M1255	GL	AVRDC, Taiwan
61	M1316	GL	AVRDC, Taiwan
62	M1334	GL	AVRDC, Taiwan
63	M1350	GL	AVRDC, Taiwan
64	M1354	GL	AVRDC, Taiwan
65	M1370	GL	AVRDC, Taiwan
66	M1372	GL	AVRDC, Taiwan
67	M1378	GL	AVRDC, Taiwan
68	M1393	GL	AVRDC, Taiwan
69	M1400	GL	AVRDC, Taiwan
70	M1429	GL	AVRDC, Taiwan
71	M1443	GL	Punjab, India
72	M1447	GL	Uttar Pradesh, India
73	M145	GL	Nwe Delhi, India
74	M1477	GL	Uttar Pradesh, India
75	M1485	GL	Maharashtra, India
76	M1493	GL	Pantnagar, India
77	M1503	GL	Andrapradesh, India
78	M186	GL	Bihar, India
79	M204	GL	Bihar, India
80	M289	GL	New Delhi, India
81	M313	GL	New Delhi, India
82	M322	GL	New Delhi, India
83	M409	GL	New Delhi, India
84	M422	GL	New Delhi, India
85	M450	GL	New Delhi, India
86	M499	GL	Uttar Pradesh, India

87	M660	GL	Punjab, India
88	M678	GL	Haryana, India
89	M684	GL	Haryana, India
90	M700	GL	New Delhi, India
91	M704	GL	New Delhi, India
92	M729	GL	Philippines
93	M739	GL	Philippines
94	M765	GL	New Delhi, India
95	M837	GL	AVRDC, Taiwan
96	M875	GL	AVRDC, Taiwan
97	M880	GL	New Delhi, India
98	M906	GL	AVRDC, Taiwan
99	M958	GL	AVRDC, Taiwan
100	M961	GL	AVRDC, Taiwan
101	M981	GL	AVRDC, Taiwan
102	M989	GL	AVRDC, Taiwan
103	MH1442	ABL	Haryana, India
104	MH215	RV	Haryana, India
105	MH318	RV	Haryana, India
106	MH565	ABL	Haryana, India
107	MH810	RV	Haryana, India
108	MH934	ABL	Haryana, India
109	MH96-1	RV	Haryana, India
110	ML1299	GL	Punjab, India
111	ML1451	GL	Punjab, India
112	ML1464	GL	Punjab, India
113	ML1628	ABL	Punjab, India
114	ML2037	GL	Punjab, India
115	ML818	RV	Punjab, India
116	MUSKAN	RV	Haryana, India
117	NM1	RV	Uttarpradesh, India
118	OLRM4	GL	Orissa, India
119	PDM139	RV	Uttar Pradesh, India
120	PLM167	GL	AVRDC, Thailand
121	PLM271	GL	AVRDC, Thailand
122	PrakashNepal	RV	Nepal
123	PS16	RV	New Delhi, India
124	PUSA0672	RV	New Delhi, India
125	PUSA0971	RV	New Delhi, India
126	PUSA1131	ABL	New Delhi, India
127	PUSA1132	ABL	New Delhi, India
128	PUSA1331	ABL	New Delhi, India
129	PUSA1332	ABL	New Delhi, India
130	PUSA1333	ABL	New Delhi, India

131	PUSA1341	ABL	New Delhi, India
132	PUSA1342	ABL	New Delhi, India
133	PUSA1441	ABL	New Delhi, India
134	PUSA871	ABL	New Delhi, India
135	PUSA9531	ABL	New Delhi, India
136	PusaBaisakhi	RV	New Delhi, India
137	PusaRatna	RV	New Delhi, India
138	PusaVishal	RV	New Delhi, India
139	RMG1028	RV	Rajasthan, India
140	RMG1087	RV	Rajasthan, India
141	RMG991	RV	Rajasthan, India
142	RMGP1	RV	Rajasthan, India
143	SATYA	RV	Haryana, India
144	TM96-2	RV	Maharashtra, India
145	TM96-25	RV	Maharashtra, India
146	TM9725	ABL	Maharashtra, India
147	V04-04	GL	AVRDC, Thailand
148	V1109	ABL	AVRDC, Thailand
149	V1138	ABL	AVRDC, Thailand
150	V1153	ABL	AVRDC, Thailand
151	V3518	ABL	AVRDC, Thailand
152	V6183	ABL	AVRDC, Thailand
153	YellowMung1	GL	West Bengal, India

(RV, released variety; ABL, advanced breeding line; GL, germplasm line)

Supplementary Table 2. Pooled ANOVA across the environments (DL and LUD)

	DF50	DF100	DM	SPAD	PL	PN	SPP	YPP	100SW	PH
G	4.49***	3.87***	2.97***	9.96***	0.41***	79.76***	0.53***	4.90***	0.11***	14.52***
E	2.64	1.06	0	0.94	1.55	5.35*	0.11	0.11	0.12	0
G x E	9.22***	2.98***	0.49*	21.07***	0	9.25**	1.11E-15	1.40E-15	0	0
Grand mean	37.02	40.33	64.03	32.14	8.28	29.07	11.10	7.68	4.85	36.78
LSD	4.33	3.31	2.44	6.60	0.74	9.45	1.01	1.55	0.47	5.11
CV	3.80	4.09	3.03	8.94	6.97	19.76	7.47	14.43	8.06	11.19

G: genotypic variance, E: environmental variance, G x E: genotypic and environmental interaction variance: LSD: least significant difference, CV: coefficient of variation, DF50: days to 50% flowering, DF100: days to 100% flowering, DM: days to maturity, SPAD: nitrogen status, PL: pod length, PN: pod number, SPP: seeds per pods, YPP: yield per plant, 100SW: 100-seed weight, PH: plant height,.***$p < 0.001$, ** $p < 0.01$ and *$p < 0.05$ level of significance.

Supplementary Table 3. Distribution of SNPs on mungbean chromosome

Chromosome number	Size of the chromosome (bp)	No. of SNPs on chromosome	Average density (SNPs per 0.1Mb)
**1**	36501346.00	2294.00	6.3
**2**	25360630.00	1400.00	5.5
**3**	12950713.00	770.00	5.9
**4**	20812224.00	1098.00	5.3
**5**	37180910.00	1495.00	4.0
**6**	37436759.00	1857.00	5.0
**7**	55601358.00	2292.00	4.1
**8**	45727239.00	1695.00	3.7
**9**	21008463.00	1264.00	6.0
**10**	20996616.00	745.00	3.5
**11**	19732206.00	1015.00	5.1
Total	**333308464.00**	**15926.00**	**54.4**

Supplementary Table 4. List of 50 significant SNPs with their respective *p* values.

Sr. No.	Env	Traits	SNPs	Chr	Position	*p* value	PVE	Effect	LOD
1	DL	DF50	S1_1401613	1	1401613	7.54E-05	0.16339	1.820905	4.122823256
2	DL	DF50	S1_1401609	1	1401609	9.53E-05	0.15538	1.798244	4.020976358
3	DL	DF50	S1_1401637	1	1401637	9.53E-05	0.15538	-1.79824	4.020976358
4	DL	DF100	S1_13315197	1	13315197	1.88E-09	0.07032	1.479525	8.72692225
5	DL	DF100	S1_1401613	1	1401613	5.73E-08	0.1857	1.457305	7.241820165
6	DL	SPAD	S1_34950474	1	34950474	7.33E-05	0.12689	-2.746	4.135070947
7	DL	SPAD	S1_34950467	1	34950467	7.70E-05	0.16403	2.782531	4.113411529
8	DL	SPAD	S1_34950502	1	34950502	7.70E-05	0.16403	2.782531	4.113411529
9	DL	SPP	S2_24801210	2	24801210	2.16E-05	0.14173	0.206612	4.665144331
10	DL	YPP	S3_7458210	3	7458210	4.40E-05	0.13793	0.881672	4.356821249
11	DL	PB	S4_16870061	4	16870061	2.37E-07	0.0951	0.18313	6.62532092
12	DL	100SW	S8_2334614	8	2334614	5.36E-05	0.18663	-0.17434	4.270981881
13	DL	100SW	S9_2808918	9	2808918	3.78E-05	0.12219	-0.12378	4.422336513
14	DL	100SW	S9_2808960	9	2808960	6.73E-05	0.11077	-0.123	4.171823384
15	DL	PB	S9_11221047	9	11221047	6.85E-11	0.27616	0.221147	10.16447846
16	DL	SPAD	S9_12665401	9	12665401	7.43E-05	0.16769	-3.20359	4.129288717
17	DL	DF50	S11_3265244	11	3265244	2.87E-05	0.1556	-1.85768	4.54178281
18	DL	DF50	S11_3265377	11	3265377	8.68E-05	0.13173	1.819192	4.061232812
19	DL	PB	S11_810817	11	810817	1.22E-07	0.08095	-0.21666	6.914119182
20	DL	PN	S11_10602411	11	10602411	7.04E-05	0.04797	6.291942	4.152301717
21	LUD	PL	S1_1260064	1	1260064	8.14E-05	0.12618	0.088336	4.089190473
22	LUD	SPP	S1_7517183	1	7517183	6.21E-05	0.1271	0.314036	4.207110492
23	LUD	YPP	S1_2065382	1	2065382	3.35E-05	0.12628	1.049634	4.474409919
24	LUD	DM	S2_21963318	2	21963318	4.52E-05	0.11927	-0.74368	4.344521721
25	LUD	DM	S2_21963356	2	21963356	4.52E-05	0.11927	-0.74368	4.344521721

26	LUD	PN	S3_7575781	3	7575781	6.33E-05	0.17252	3.914049	4.19839981
27	LUD	DF100	S7_4407415	7	4407415	6.12E-05	0.13097	-1.3127	4.213363845
28	LUD	PH	S7_33956225	7	33956225	6.34E-05	0.13971	0.545324	4.197810736
29	LUD	SPP	S7_53198193	7	53198193	8.07E-05	0.12485	-0.2844	4.093325145
30	LUD	PL	S8_209897	8	209897	1.23E-05	0.16572	0.118123	4.90968026
31	LUD	PL	S8_13451256	8	13451256	1.64E-05	0.13834	-0.12098	4.785668127
32	C BLUP	100SW	S1_23992177	1	23992177	8.09E-05	0.15302	0.161901	4.091848474
33	C BLUP	PH	S1_33479087	1	33479087	8.47E-05	0.15217	-1.66843	4.072281158
34	C BLUP	PH	S1_33479088	1	33479088	8.47E-05	0.15217	-1.66843	4.072281158
35	C BLUP	YPP	S1_2065382	1	2065382	8.31E-05	0.11006	0.978036	4.080232352
36	C BLUP	DM	S2_21963318	2	21963318	5.21E-05	0.12397	-0.87261	4.282948308
37	C BLUP	DM	S2_21963356	2	21963356	5.21E-05	0.12397	-0.87261	4.282948308
38	C BLUP	PL	S2_436374	2	436374	5.80E-05	0.13322	-0.2611	4.236358074
39	C BLUP	SPP	S2_24810267	2	24810267	9.30E-05	0.14601	0.297002	4.031569385
40	C BLUP	PN	S3_7458210	3	7458210	5.07E-05	0.13803	4.190066	4.294618619
41	C BLUP	PL	S5_32684304	5	32684304	4.93E-06	0.13656	-0.27801	5.306899136
42	C BLUP	PL	S7_55475454	7	55475454	2.64E-05	0.12987	0.329244	4.577627255
43	C BLUP	PL	S7_33485549	7	33485549	5.10E-05	0.15434	-0.27111	4.292489469
44	C BLUP	PL	S7_55475382	7	55475382	5.64E-05	0.12051	-0.31253	4.249013883
45	C BLUP	100SW	S8_2334614	8	2334614	4.96E-05	0.20374	-0.17977	4.304543915
46	C BLUP	PL	S8_13451256	8	13451256	9.48E-06	0.13558	-0.34448	5.02333871
47	C BLUP	PL	S8_209897	8	209897	2.05E-05	0.13209	0.320249	4.688467318
48	C BLUP	SPAD	S8_38348926	8	38348926	2.82E-05	0.15159	0.953522	4.550264815
49	C BLUP	100SW	S9_2808918	9	2808918	3.88E-05	0.12425	-0.12691	4.410679916
50	C BLUP	100SW	S9_2808960	9	2808960	7.95E-05	0.11209	-0.12514	4.099381041

Env: environments, Chr: chromosomes, DL: Delhi, LUD: Ludhiana, C BLUP: Combined Best Linear Unbiased Predictors, PVE: phenotypic variance explained, DF50: days to 50% flowering, DF100: days to 100% flowering, DM: days to maturity, SPAD: nitrogen status, PH: plant height, PB: primary branch, PL: pod length, PN: pod number, SPP: seeds per pod, 100SW: 100-seed weight and YPP: yield per plant.

Supplementary Table 5. Soil analysis data of Delhi and Ludhiana locations

Soil analysis data of different locations	Delhi		Ludhiana	
	R_1	R_2	R_1	R_2
pH	8.51	8.45	7.56	7.51
ES (ds/m)	0.38	0.34	0.51	0.50
Organic Carbon Content (%)	0.44	0.46	0.54	0.51
Available N (kg/ha)	184	180	153	151
Available P (kg/ha)	25.5	25.7	16.2	16.9
Available K (kg/ha)	290	286	199	203
Texture	Sandy loam	Sandy loam	Sandy loam	Sandy loam
Soil Fe concentration (mg/kg)	5.01	4.96	8.3	8.1
Soil Zn concentration (mg/kg)	1.68	1,67	0.75	0.79

Supplementary Figure 1. Correlation coefficients and level of significance for phenological and agronomic traits of 153 mungbean genotypes. Figure 2(a) shows a correlation of traits at Delhi environment. Here, maximum traits showed positive correlation with yield and among themselves, except negative correlation was observed between traits DF100 and PH ($p<0.05$). Figure 2(b) shows correlation of traits at Ludhiana environment. Here also similar results were found except traits PH had shown negative correlation with traits DF50, DF100, DM ($p<0.001$) and PB ($p<0.01$).

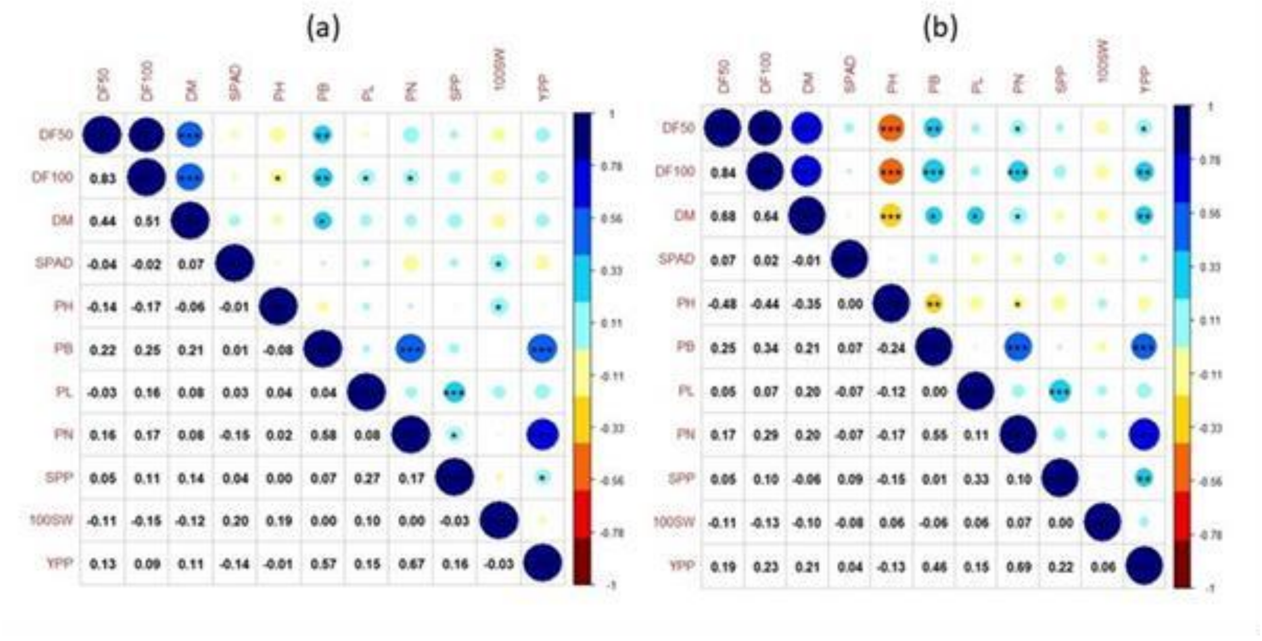

Supplementary Figure 2. Distribution of SNPs over 11 different chromosomes of mungbean after identification of SNPs by GWAS. Chromosome 1 shows the highest number of SNPs whereas Chromosome 10 shows the lowest number of SNPs.

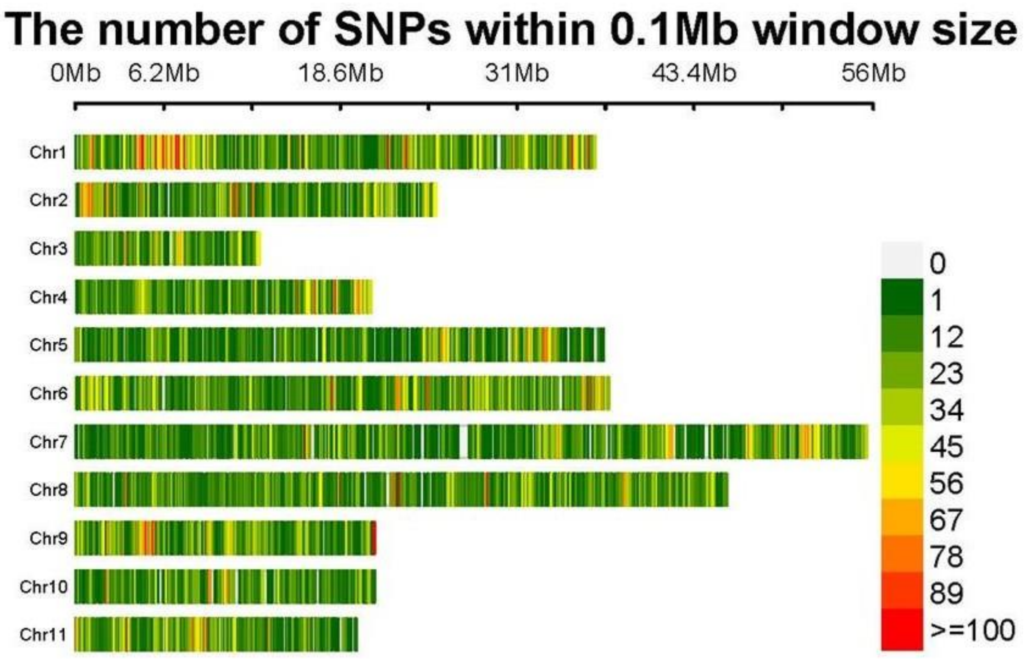

Supplementary Figure 3: Digital gene expression studies of 19 candidate genes positively or negatively correlated with yield in mungbean. The colour codes are given for the intensity of their expressions. The red colour indicates high expressions, the brown is moderate and the yellow indicates low expression

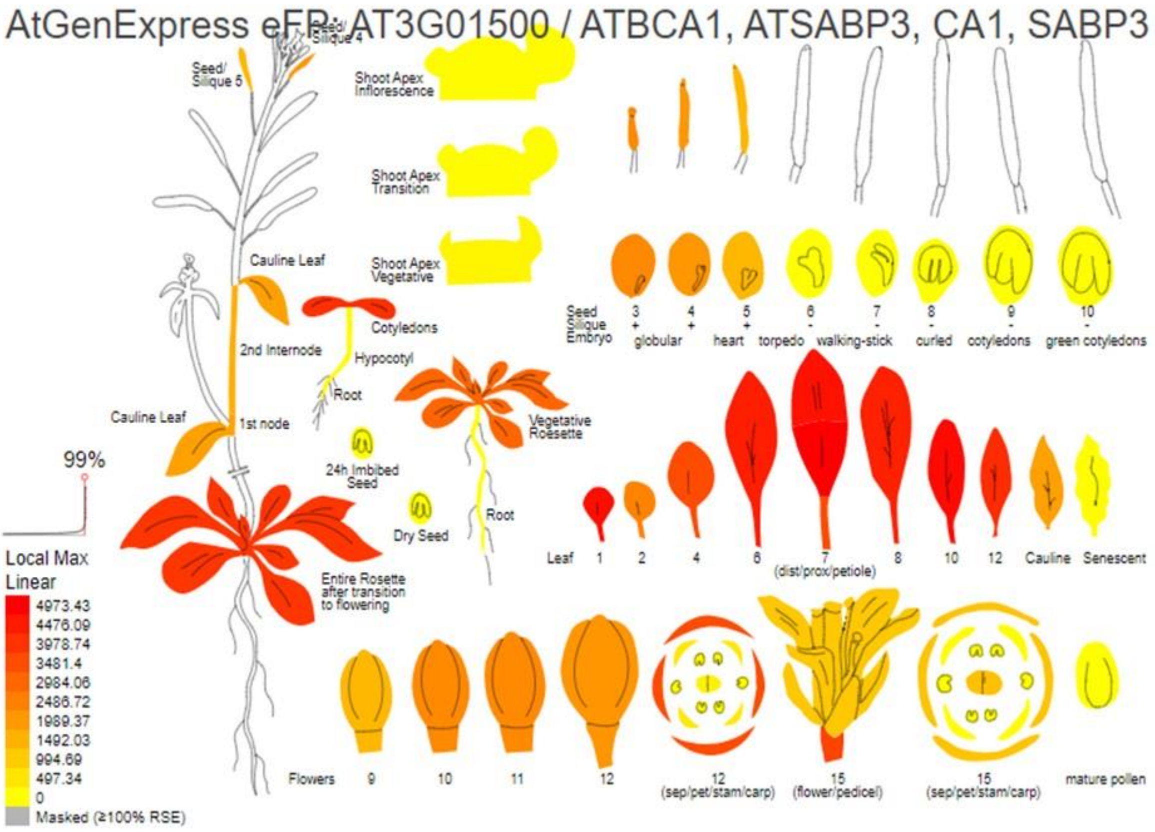

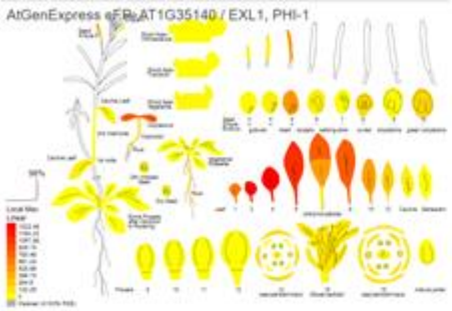
AtGenExpress eFP: AT1G35140 / EXL1, PHI-1

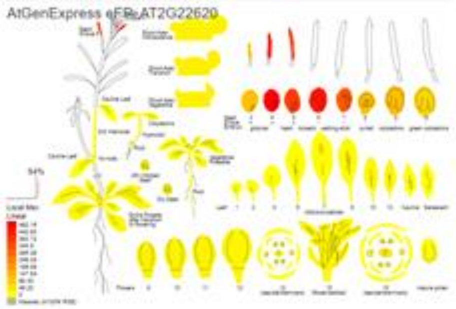
AtGenExpress eFP: AT2G22620

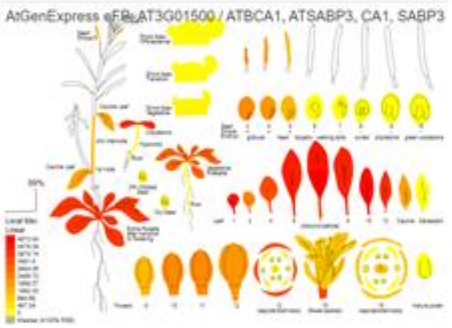
AtGenExpress eFP: AT3G01500 / ATBCA1, ATSABP3, CA1, SABP3

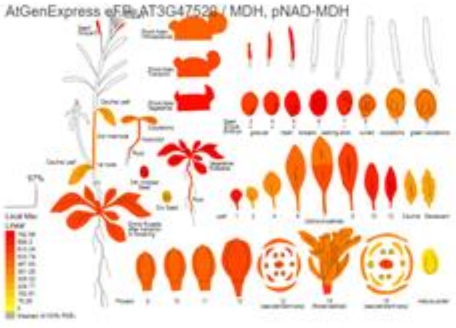
AtGenExpress eFP: AT3G47520 / MDH, pNAD-MDH

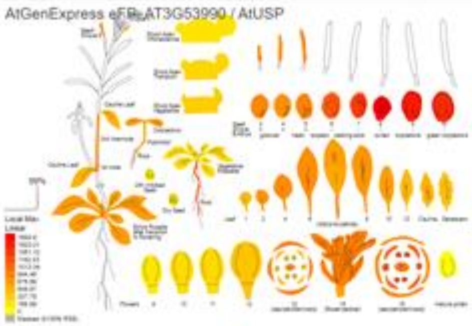
AtGenExpress eFP: AT3G53990 / AtUSP

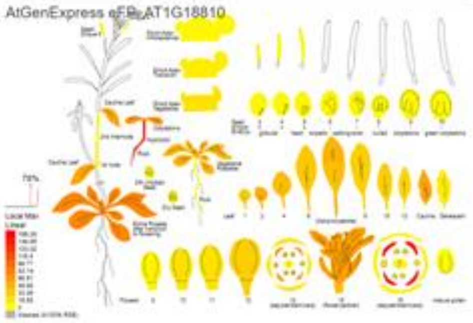
AtGenExpress eFP: AT1G18810

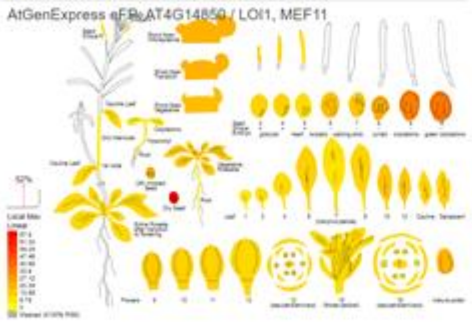
AtGenExpress eFP: AT4G14850 / LOI1, MEF11

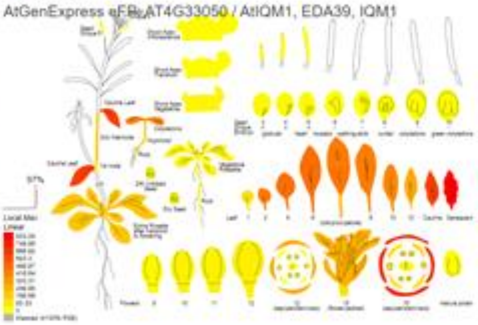
AtGenExpress eFP: AT4G33050 / AtIQM1, EDA39, IQM1

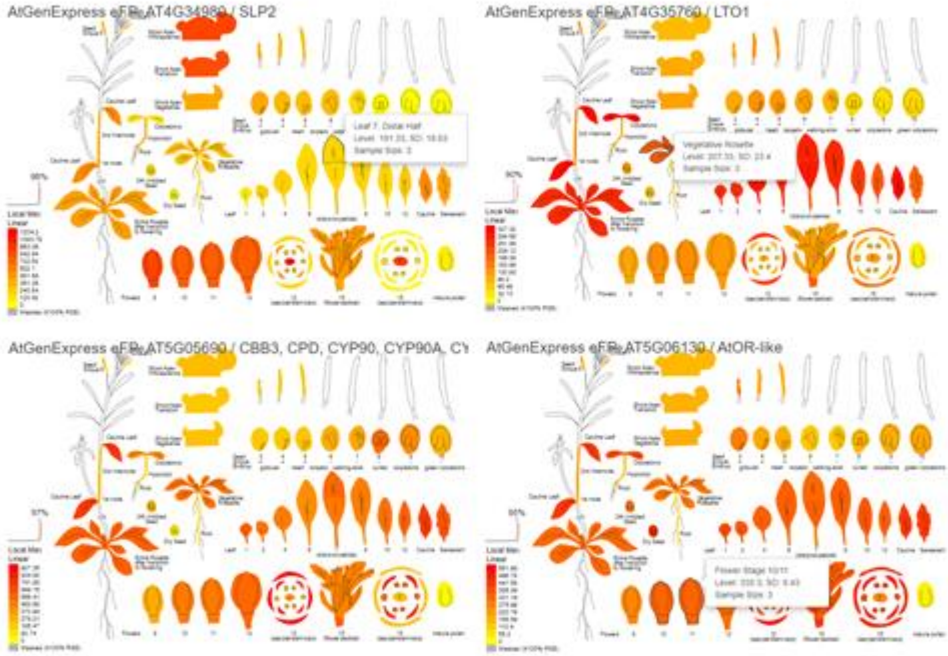
AtGenExpress eFP: AT4G34980 / SLP2
AtGenExpress eFP: AT4G35760 / LTO1
AtGenExpress eFP: AT5G05690 / CBB3, CPD, CYP90, CYP90A, C
AtGenExpress eFP: AT5G06130 / AtOR-like

.
